# Construction of hyperthermostable d‐allulose 3‐epimerase from *Arthrobacter globiformis* M30 using the sequence information from *Arthrobacter psychrolactophilus*

**DOI:** 10.1002/2211-5463.70060

**Published:** 2025-06-09

**Authors:** Kensaku Shimada, Kouhei Ohtani, Pushpa Kiran Gullapalli, Kazuhiko Ishikawa

**Affiliations:** ^1^ Matsutani Chemical Industry Co., Ltd. Itami Hyogo Japan

**Keywords:** chimera, isomerization, protein engineering, psicose, rare sugar, stability

## Abstract

d‐Allulose is one of the rare monosaccharides and is considered as a safe ingredient in foods. It can be enzymatically produced from d‐fructose by the enzyme d‐allulose 3‐epimerase. More stable enzymes can operate effectively for longer durations, reducing the need for frequent replacements and thereby lowering costs. In addition, the preparation of the recombinant *Arthrobacter globiformis* M30 (AgDAE) enzyme requires heat treatment at 60–70 °C to remove host cell debris and potential microbial contaminants. Therefore, to address the need for more thermostable enzymes in d‐allulose production, we aimed to create thermostable mutants of AgDAE using the protein engineering method. We cloned d‐allulose identified from *A. globiformis* M30 and, using sequence homology, we constructed thermostable mutants by protein engineering. Each effect of the five mutations used was independent and additive. By integrating positive mutations, we succeeded in the construction of a chimeric enzyme exhibiting hyperthermostability without loss of enzymatic activity. The constructed chimera mutant was highly functional above 95 °C and remained stable under 80 °C. Our approach using structural information for the chimeric construction experiments also suggested that incorporating mutations from other homologous enzymes can impart advantages in enzymes in a simple and effective manner.

## Graphical abstract

d‐Allulose can be produced from d‐fructose by d‐allulose 3‐epimerase. Based on sequence homology information, we successfully engineered thermostable mutants with the protein engineering method. By integrating positive mutations, we constructed an enzyme that exhibits hyperthermostability without a loss in the activity. The resulting mutant remains highly functional at temperatures above 95 °C and stable at temperatures below 80 °C (○, Wild; ●,Mutant_5m; □, Mutant_E).

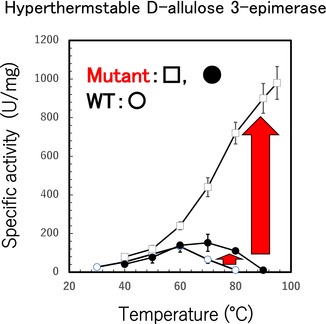

**Abbreviations**
AgDAE: d‐allulose 3‐epimerase from *Arthrobacter globiformis* strain M30
ApDAE: d‐allulose 3‐epimerase from *Arthrobacter psychrolactophilus*
AtDAE: d‐allulose 3‐epimerase from *Agrobacterium tumefaciens*
CcDAE: d‐allulose 3‐epimerase from *Clostridium cellulolyticum*
DAE: d‐allulose 3‐epimerase
MlLRE: l‐ribulose 3‐epimerase from *Mesorhizobium loti*
PcDTE: d‐tagatose 3‐epimerase from *Pseudomonas cichorii*
WT: wild‐type

d‐allulose, also known as d‐psicose, is a low‐calorie sweetener that is considered a promising alternative to traditional sugars [1]. Physiological functions of d‐allulose, such as moderating blood glucose levels and reducing fat accumulation, have attracted more and more attention in the field of human healthcare [2, 3, 4]. d‐allulose has been accepted as Generally Recognized as Safe (GRAS; GRAS Notices 498 and others) by the US Food and Drug Administration, and is also used as a safe food ingredient in Japan, Mexico, South Korea and other countries. In the early 1990s, pilot‐scale production of d‐allulose from d‐fructose by d‐tagatose 3‐epimerase was first reported [5]. It was later found that d‐allulose 3‐epimerase from *Arthrobacter globiformis* M30 (AgDAE) can effectively produce d‐allulose from d‐fructose [6]. The first crystal structure of d‐allulose 3‐epimerase (also known as d‐psicose 3‐epimerase) from *Agrobacterium tumefaciens* (AtDAE) has been determined, and the data have been deposited in the Protein Data Bank as pdb:2hk0 [7]. The enzyme is a tetramer and each monomer belongs to a TIM‐barrel fold. The active site contains a metal ion coordinated in an octahedral arrangement with two water molecules and four residues that are highly conserved within the family. Structural evidence and site‐directed mutagenesis experiment suggest that the metal ion plays an important role in catalysis by anchoring the bound substrate, and the conserved Glu150 and Glu244 are catalytic residues for epimerization reaction at the C‐3 position of substrate. The substrate displaces water molecules in the active site, with a conformation mimicking the intermediate cis‐enediolate [7]. From the sequence homology, it has been assumed that the structures and reaction mechanisms between AgDAE and AtDAE are similar.

Recently, Matsutani Chemical Industry Co., Ltd (Hyogo, Japan) has developed a mass production system for d‐allulose and commercialized it under the brand name Astraea, utilizing the enzyme AgDAE. To produce d‐allulose industrially at 50 °C, thermostable enzymes are essential because they can remain stable and functional for extended periods at this temperature. The stability of these enzymes directly influences the overall cost of d‐allulose production. More stable enzymes can operate effectively for longer durations, reducing the need for frequent replacements and thereby lowering costs.

In addition, the preparation of the recombinant enzyme AgDAE requires heat treatment at 60–70 °C to remove host cell debris and potential microbial contaminants. Therefore, developing a more hyperthermostable AgDAE enzyme is critical. Enhancing the thermostability of AgDAE would improve the efficiency of the d‐allulose production process, ultimately reducing production costs and meeting the growing industrial demand for stable enzymes. To address the need for thermostable enzymes in d‐allulose production, we are working on creating thermostable mutants of AgDAE using the protein engineering method.

One approach that we frequently employ is random mutagenesis, such as error‐prone PCR [8], to enhance enzyme thermostability. Additionally, computational tools such as foldx [9] and rosetta [10] are being developed to aid in the design of thermostable enzymes. However, it remains unclear whether the mutation site predictions generated by these computational methods are more accurate than those derived from random based approaches [9]. Achieving a rational design for thermostable enzymes that maintains specific activity is an important goal for basic research and industrial applications. Nonetheless, there is currently no widely accepted and effective strategy for efficient enzyme engineering.

We have successfully obtained several thermostable mutants of AgDAE [11] by the sequence shuffling method [12] with protein engineering. Meanwhile, a thermostable d‐allulose 3‐epimerase from *Arthrobacter psychrolactophilus* (ApDAE) has been cloned and characterized [13]. From the high sequence homology between these enzymes, it has been predicted that their tertiary structures and active sites are quite similar. By analyzing the sequence homology between AgDAE and ApDAE, alongside the crystal structure of AtDAE [7], we aimed to identify stable structural regions of ApDAE. This led us to attempt creating a hyperthermostable AgDAE by constructing a chimera enzyme that combines select parts of both AgDAE and ApDAE.

## Materials and methods

d‐allulose (Astraea) was prepared by Matsutani Chemical Industry Co., Ltd. Peptone (Nucel 581PW) and yeast extract (BSP‐B) were purchased from Oriental Yeast (Tokyo, Japan). Other chemicals were purchased from Nacalai Tesque Inc. (Kyoto, Japan). The protein purification column (Superdex™ 200 increase 10/300 GL) was purchased from Cytiva (Tokyo, Japan).

### Enzyme assay

**Fig. 1:**
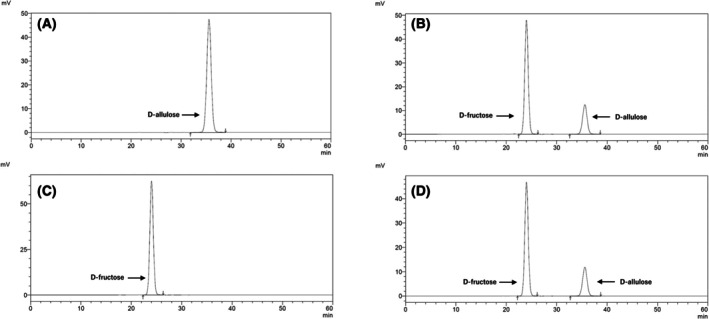
Chromatograms of the epimerization reaction. (A) Chromatogram of forward reaction solution in which d‐allulose was not reacted with the enzyme (substrate blank). (B) Chromatogram of forward reaction solution in which d‐allulose reacted with the enzyme (substrate + enzyme). (C) Chromatogram of reverse reaction solution in which D‐fructose did not react with the enzyme (substrate blank). (D) Chromatogram of reverse reaction solution in which D‐fructose reacted with the enzyme (substrate + enzyme).

the d‐allulose 3‐epimerase (DAE) activity assay was performed by the method based on the quantitative analysis by determining the amount of d‐fructose produced from d‐allulose [11]. The reaction mixture contained 2 mm MgSO_4_ and 100 mm d‐allulose in 50 mm phosphate buffer (pH 8.0), to which an appropriate amount of diluted enzyme was added, with a final volume of 1.0 mL. The reaction mixture was incubated at 50 °C for 10 min and the reaction was stopped by adding 0.04 mL of 5% HCl solution. The amount of d‐fructose produced was determined using a HPLC analysis system [6] equipped with MCI GEL CK08EC column from Mitsubishi Chemical (Tokyo, Japan) and refractive index detector. The injection volume was 10 μL, distilled water was used as the mobile phase at flow rate of 0.4 mL·min^−1^ and the column temperature was maintained at 80 °C. An example of the analysis data obtained by HPLC is shown in Fig. 1. The control sample without enzyme consistently shows no peak for the product, serving as a reliable blank. To ensure accurate enzyme activity measurement, the reaction was conducted under conditions where the reaction ratio remained within 10%. Under these conditions, the amount of product formed is proportional to time. The initial velocity determined from the slope of the product formation curve represents the enzyme activity. Therefore, the enzyme concentration was adjusted to maintain the reaction ratio within 10% for precise activity assays. One unit of enzyme activity was defined as the amount of enzyme that could epimerize 1 μmol of substrate in 1 min. The values of *k*_cat_ and *K*_m_ for the substrates were determined by the least squares method [14] for Michaelis–Menten equation at 50 °C in 50 mm sodium phosphate buffer (pH 8.0) containing 2 mm MgSO_4_. For the reverse reaction (d‐fructose → d‐allulose), d‐allulose produced from d‐fructose was also determined by HPLC with the same method described above. Protein concentrations were determined using the Bradford protein assay kit (Bio‐Rad Laboratories, Berkeley, CA, USA) with BSA as the standard protein.

### Enzyme preparation and purification

The structural genes encoding AgDAE [6] and ApDAE [13] were prepared by chemical synthesis. The preparation and purification of the recombinant enzymes were carried out as described previously [11]. The purity of the enzymes was checked by SDS/PAGE. The constructed structural genes of AgDAE and ApDAE amplified by PCR were fused with *Nco*I digested pQE60 using an In‐Fusion HD Cloning Kit (Takara Bio Inc., Shiga, Japan). Using the plasmid, the transformant *Escherichia coli* M15 was prepared and the recombinant enzymes were expressed. *Escherichia coli* harboring the recombinant enzyme was cultivated at 37 °C for 6 h in SB medium (3.2% Peptone Nucel 581PW, 2.0% Yeast extract BSP‐B and 0.5% NaCl) containing 0.01% ampicillin and 0.005% kanamycin. Enzyme expression was induced by the addition of isopropyl thio‐β‐d‐galactoside to the medium, resulting in a final concentration of 0.1 mm isopropyl thio‐β‐d‐galactoside and cultivation was continued at 20 °C for overnight. The cells were then harvested by centrifugation and the collected cells were suspended in 50 mm sodium phosphate buffer (pH 8.0) containing 2 mm MgSO_4_ and disrupted by sonication for 15 min. The resulting solution was heated at 55 °C for 15 min, and cell debris was removed by centrifugation. DNA present in the cell free extract was removed using a Nucleic Acid Removal Kit (ProFoldin, Hudson, MA, USA). The supernatant was applied onto a purification column (Superdex™ 200 increase 10/300 GL column; Cytiva, Marlborough, MA, USA). The active fractions were pooled and used for the experiments. The purity and molecular weight of the sample was determined by SDS/PAGE. The protein sample was mixed with an Invitrogen NuPAGE™ LDS Sample Buffer (4×) and NuPAGE™ Reducing Agent (10×) (Thermo Fisher Scientific, Waltham, MA, USA) to adjust the final concentration to 0.2 mg·mL^−1^. After heating at 70 °C for 10 min, each 5 μL of the prepared solution was loaded onto the SDS polyacrylamide gel, as reported previously [6]. All mutant genes were constructed by PCR using a PrimeSTAR Max DNA Polymerase (Takara Bio Inc.) and the recombinant mutant enzymes were also prepared and purified, as described above.

### Thermostability of the enzymes

Thermostability of the mutant enzymes were determined as the melting temperature value (*T*_m_) using a Protein Thermal Shift Dye Assay kit (TSA; Thermo Fisher Scientific) [12]. The individual mutant samples were dissolved in 50 mm sodium phosphate buffer (pH 8.0) containing 2 mm MgSO_4_. The dye from the assay kit binds to exposed internal hydrophobic amino acid residues of the enzyme allowing the thermal stability of proteins to be monitored using a real‐time PCR (Applied Biosystems, Foster City, CA, USA). Measurement was performed according to the PCR System analysis software and assay kit user manual. The residual activity assay of the enzyme after heat treatment was performed using the method based on the quantitative analysis by determining the amount of d‐fructose produced from d‐allulose at 50 °C with pH 8.0 as described above after incubation at 80 °C with pH 8.0. Optimum temperatures of the enzymes were determined in the same condition as described above.

## Results and Discussion

### Validation of the effectiveness of the sequence shuffling method for DAE

**Fig. 2:**
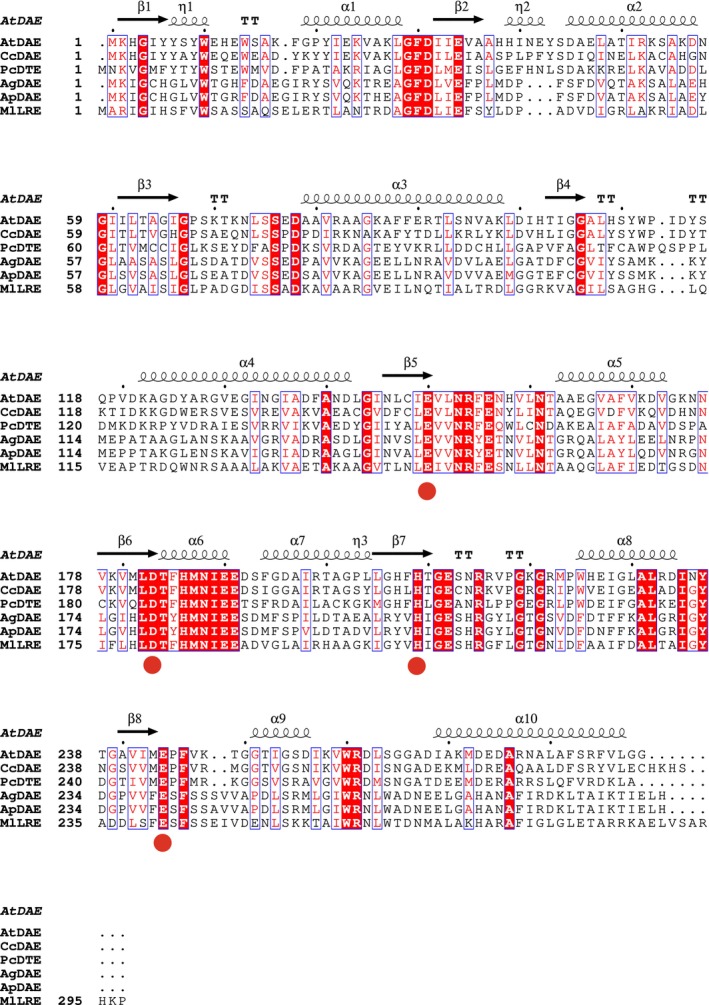
Multiple sequence alignment of DAE and its homologs. The sequences for DAE from *A. globiformis* M30 (AgDAE; GenBank accession no: AB981957) [6] were aligned with those from *A. psychrolactophilus* (ApDAE: GenBank accession no: PYI39447), *A. tumefaciens* (AtDAE; GenBank accession no: AAL45544), *C. cellulolyticus* (CcDAE; GenBank accession no: ACL75304), *P. cichorii* (PcDTE; GenBank accession no: BAA24429) and *Mesorhizobium loti* (MlLRE; GenBank accession no: BAB50266). The residues in red enclosed in a blue box are highly conserved and those with red background are identical. The speculated secondary structure elements are shown by the structural information of AtDAE [7]. The conserved catalytic residues coordinating the metal ion are indicated by red circles. The mutated amino acids in AgDAE_5m are indicated in cyan. Multiple sequence alignment figure was prepared using ESPrint3.0.

Many types of DAEs have been found, cloned and characterized. It was reported that DAE from *A. globiformis* exhibits high activity [6] and its thermostable mutants were constructed rationally [11] using the structural information by alphafold2 [15]. The rational design for thermostable AgDAE without sacrificing its specific activity was carried out [11] by the sequence shuffling method [12]. We succeeded in the construction of thermostable mutants of AgDAE by the five point mutations (AgDAE_5m: Glu75Pro, Ser137Lys, Ala200Lys, Val237Ile and Ala270Lys) with the protein engineering method (Table 1) [11]. For Glu75Pro, we could stabilize the loop region by the introduction of Pro [11, 16, 17, 18]. For Val237Ile, we could improve the molecular packing and/or hydrophobic interaction [11, 12, 18]. For Ser137Lys, Ala200Lys and Ala270Lys, we could strengthen the protein fold by the introduction of salt bridge at the surface of the enzyme [11, 19, 20, 21]. It was reported that AgDAE_5m ultimately achieved an approximately 12 °C increase in thermostability [11]. Until the present study, AgDAE_5m was the most thermostable mutant enzyme among AgDAE derivatives examined. Recently, thermostable DAE from *A. psychrolactophilus* (ApDAE) has been cloned and characterized [13]. Figure 2 shows the sequence alignment of DAEs; AtDAE [22], ApDAE [13], d‐allulose 3‐epimerase from *Clostridium cellulolyticum* (CcDAE), d‐tagatose 3‐epimerase from *Pseudomonas cichorii* (PcDTE), AgDAE [6] and l‐ribulose 3‐epimerase from *Mesorhizobium loti* (MlLRE). The enzyme exhibits a tetramer structure and each monomer belongs to a TIM‐barrel fold. The active site contains a metal ion coordinated in an octahedral arrangement with two water molecules and four residues that are highly conserved within in the family (Fig. 2). Structural evidence [7] and site‐directed mutagenesis experiment suggest that the metal ion plays an important role in catalysis by anchoring the bound substrate, and the conserved Glu150 and Glu244 are catalytic residues for the epimerization reaction of AgDAE. From the sequence homology, it has been assumed that the structures and reaction mechanisms between AgDAE and AtDAE are similar. AgDAE exhibits high sequence homology (88.2%) with ApDAE. To compare the characteristics between AgDAE and ApDAE, we prepared the recombinant ApDAE. The enzyme was successfully expressed, and its characteristics were subsequently analyzed. As reported previously [13], ApDAE exhibited higher activity and thermostability than those of AgDAE (Table 1). Although crystal structural information is unavailable for both AgDAE and ApDAE, their predicted tertiary structures and active sites based on sequence homology suggest they are quite similar within the family. From the structural similarity, it can be predicted that the catalytic residues (Glu146, Asp179, His205 and Glu240) coordinating the metal ion of AgDAE are well conserved in ApDAE. Furthermore, most of the residues that relate to the enzyme activity are also well conserved. Therefore, the structural model of ApDAE predicted by alphafold2 [15] would provide the chance for the construction of the more thermostable ApDAE mutant by the rational design strategy used for the thermostabilized mutant (AgDAE_5m) described previously [11]. Based on the sequence and structural homology between ApDAE and AgDAE, it was clarified that the five sites (Glu75, Ser137, Ala200, Val237 and Ala270), which are mutation sites for the construction of AgDAE_5m, are structurally conserved in ApDAE (Fig. 2). Therefore, the same strategy for AgDAE can be applied when designing thermostable mutations for ApDAE. We constructed the corresponding mutant (ApDAE_5m: Glu75Pro, Ala137Lys, Val200Lys, Val237Ile and Ala270Lys) with the protein engineering method. It was confirmed that the recombinant ApDAE_5m was successfully expressed, and its thermostability increased by approximately 5 °C without compromising the enzyme activity (Table 1), With these five point mutations, *T*_m_ values of AgDAE and ApDAE were increased by approximately 12 °C (AgDAE_5m) and 5 °C (ApDAE_5m), respectively (Table 1). These results indicate that the level of thermostability enhancement varies between the two enzymes but the employed strategy for the construction of more thermostable enzyme (introduction of Pro residue, enhancement of molecular packing/hydrophobic interaction and introduction of salt bridge) are effective and can be applied to other DAEs.

**Table 1:** Characteristics of AgDAE, ApDAE and their mutants. The enzyme activities were measured at 50 °C in 50 mm sodium phosphate buffer (pH 8.0) containing 2 mm MgSO_4_. The concentration of each enzyme is 2.0 μg·mL^−1^ and the substrate concentration is 100 mm. The unit was determined in triplicate with the SD. Relative activity was set to 100% with wild‐type (WT) as the reference. The values of *T*_m_ (°C) were measured by TSA.

	Specific activity (U·mg^−1^)	Relative (%)	*T*_m_ (°C)
AgDAE [11]	93.0 ± 9	100	66.60
AgDAE_5m [11] (E75P/S137K/A200K/V237I/A270K)	76.3 ± 15	82	78.90
ApDAE	118.0 ± 12	127	86.45
ApDAE_5m (E75P/A137K/V200K/V237I/A270K)	99.5 ± 11	107	91.20
AgDAE_5m [11] (E75P/S137K/A200K/V237I/A270K)	76.3 ± 15	82	78.90
Mutant_A (AgDAE_5m + H13R/S245A)	96.7 ± 16	104	79.93
Mutant_B (AgDAE_5m + A117P/A120K/A123E/A128V/V129I/V132I/D138G)	83.9 ± 10	90	77.14
Mutant_C (AgDAE_5m + V34I/P172G/I176V/S218N/T223N)	78.6 ± 13	85	94.57
Mutant_D (AgDAE_5m + Mutant_A + B + C)	83.9 ± 9	90	93.54
Mutant_E (AgDAE_5m + Mutant_A + C)	120.0 ± 28	129	96.08

### Design of hyper‐thermostable DAE by chimeric sequence shuffling method (ApDAE and AgDAE_5m)

**Fig. 3:**
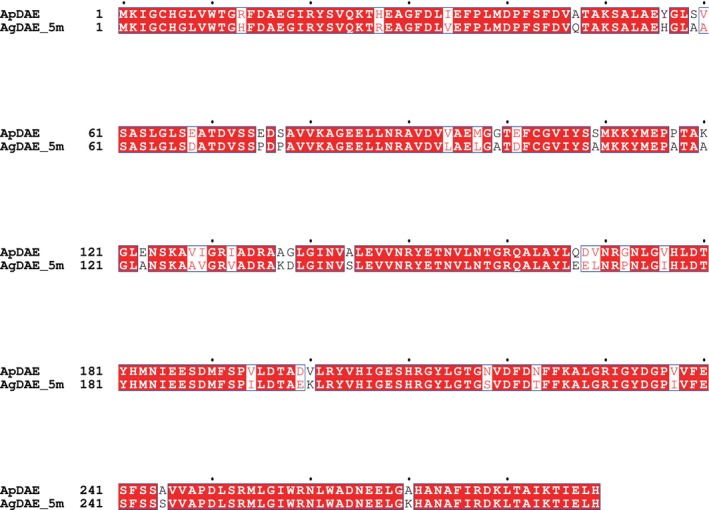
The sequence homology between ApDAE and AgDAE_5m. The residues in red are conserved between them. The mutated amino acids for AgDAE_5m are indicated in cyan. The figure was prepared by ESPript3.0.

**Fig. 4:**
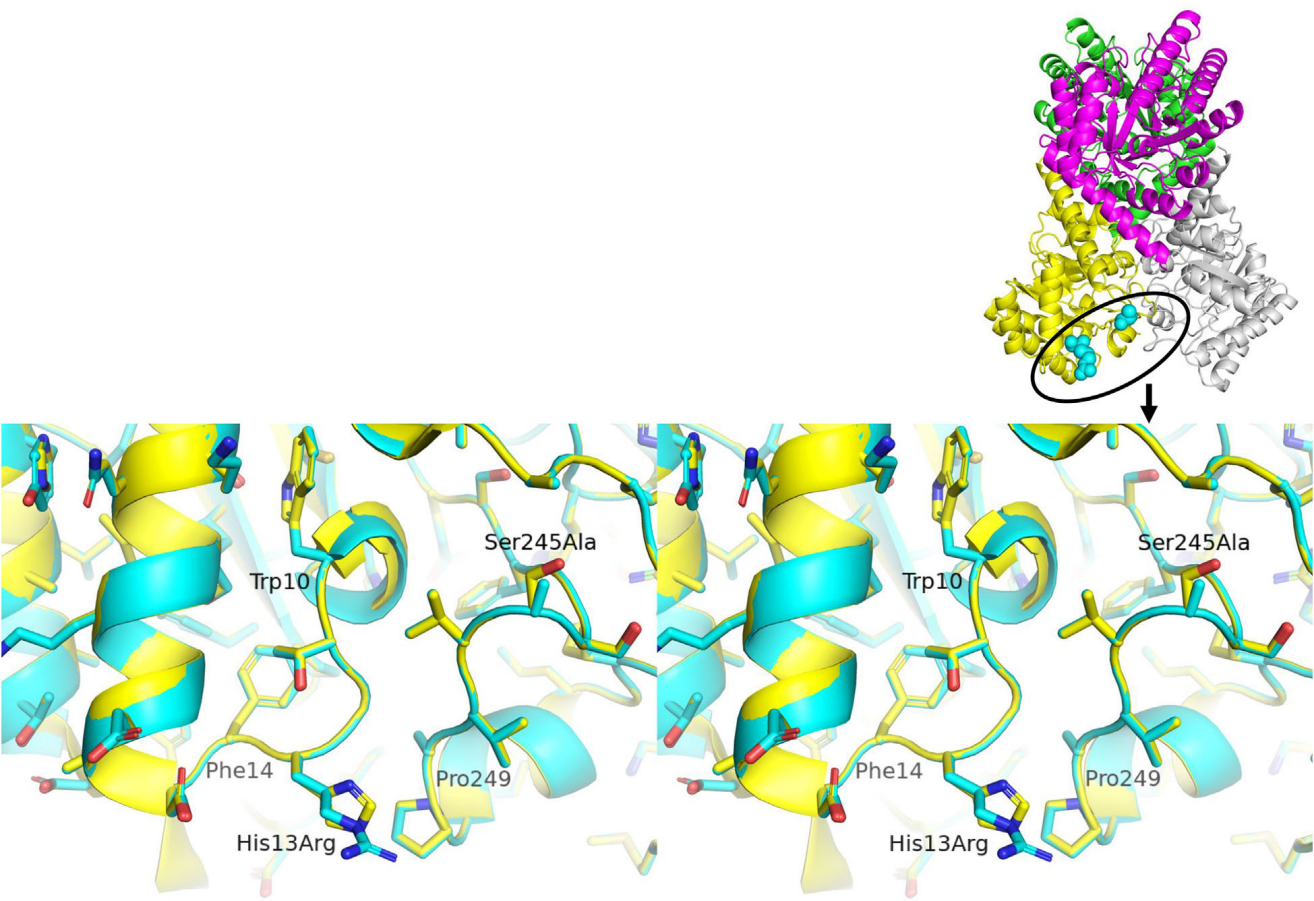
Cross‐eyed stereo figures of the superimposed two model structures of ApDAE and AgDAE_5m for Mutant_A (cyan: ApDAE; yellow: AgDAE_5m). Inset: an outline of the mutation sites introduced in homo‐tetramer structural model of AgDAE_5m. For Mutant_A, mutation sites are observed on the surface in each molecule within the tetrameric structure. The portion is zoomed in cross‐eyed stereo figures of the superimposed two model structures for Mutant_A (cyan: ApDAE; yellow: AgDAE_5m). Mutation site for His13Arg and Ser245Ala are located in two individual loops (10–14 and 245–249). The structural model was predicted by alphafold2 [15] and the figures were prepared using PyMOL [23].

**Fig. 5:**
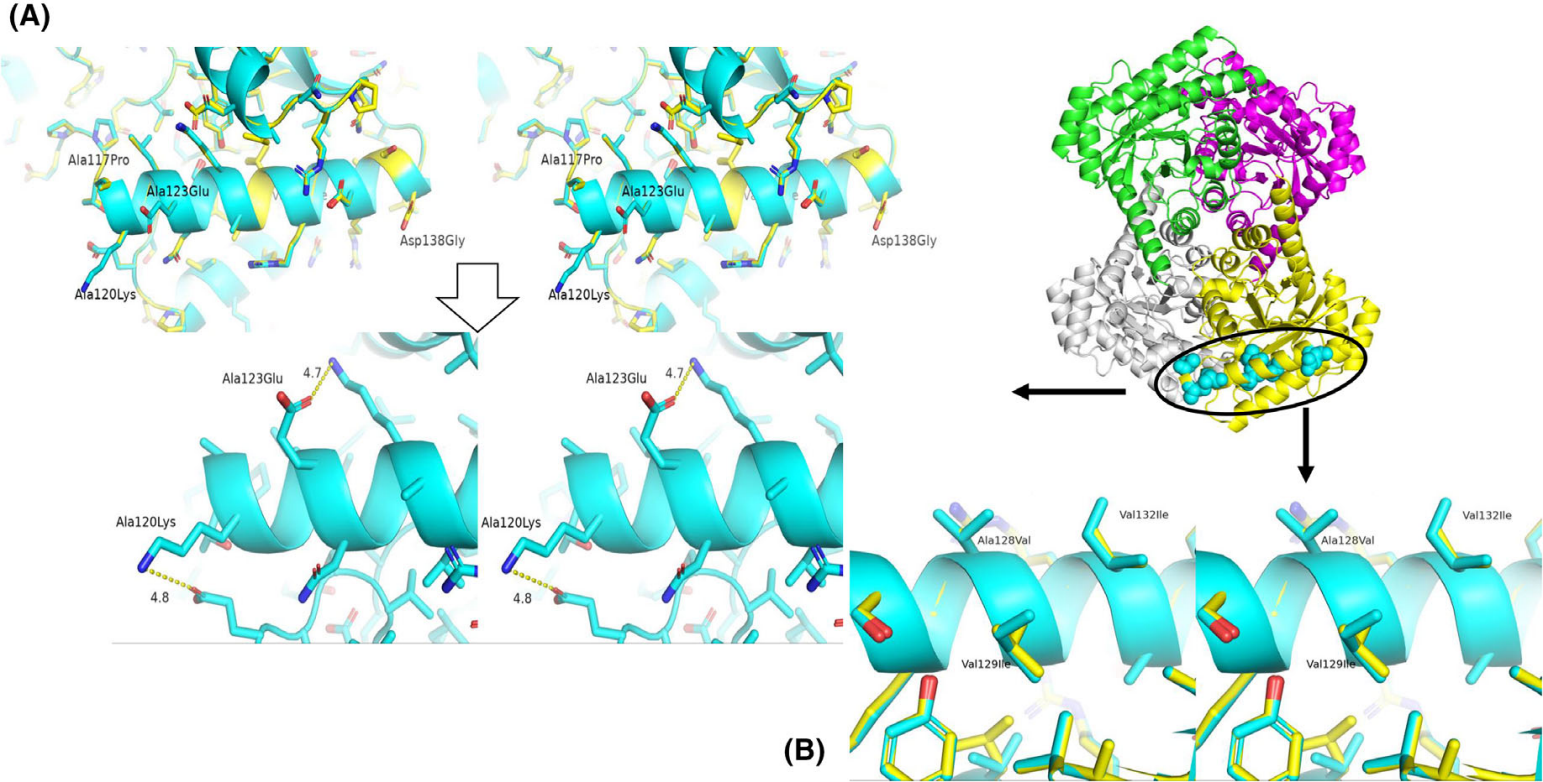
Cross‐eyed stereo figures of the superimposed two α4 helix model structures of ApDAE and AgDAE_5m for Mutant_B (cyan: ApDAE; yellow: AgDAE_5m). Inset: an outline of the mutation sites introduced in homo‐tetramer structural model of AgDAE_5m. For Mutant_B, mutation sites are observed in α4 helix structure of each molecule within the tetrameric structure. A portion is zoomed in cross‐eyed stereo figures of the superimposed two α4 helix model structures of ApDAE and AgDAE_5m for Mutant_B (cyan: ApDAE; yellow: AgDAE_5m). (A) Mutation sites in α4 helix (A117P/A120K/A123E/D138G). The dashed lines represent the predicted salt bridges for Lys120 and Glu123 in ApDAE. (B) Mutation sites in α4 helix (A128V/V129I/V132I). The structural model was predicted by alphafold2 [15] and the figures were prepared using PyMOL [23].

**Fig. 6:**
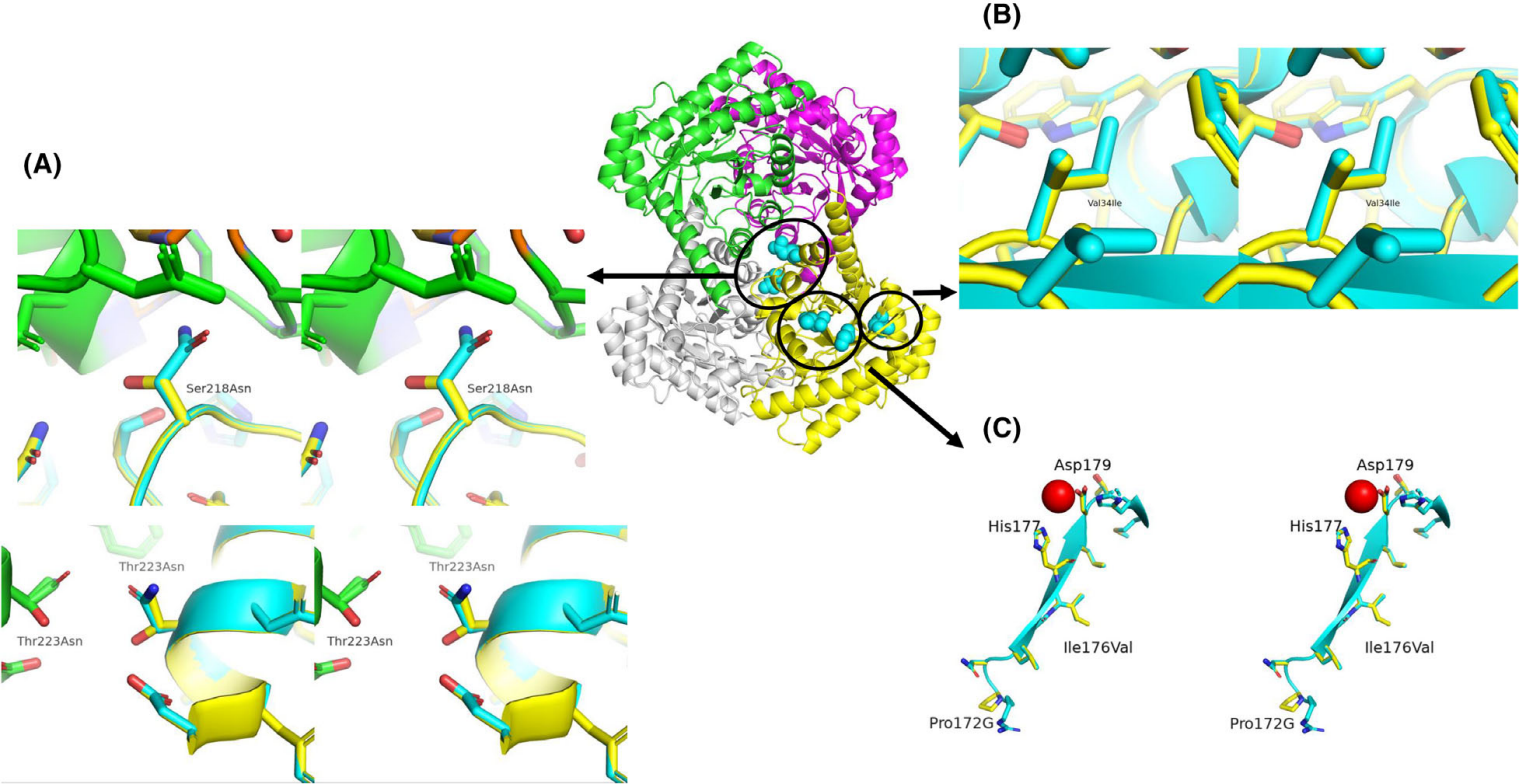
Cross‐eyed stereo figures of the superimposed two model structures for Mutant‐C (cyan: ApDAE; yellow: AgDAE_5m). Inset: an outline of the mutation sites introduced in homo‐tetramer structural model of AgDAE_5m is shown in the inset. (A) Mutation site at Ser218Asn and Thr223Asn. Models shown in green represent the another molecule in tetramer structure of the enzyme. (B) Mutation site at Val34Ile. (C) Mutation sites at Pro172Gly/Ile176Val. His177 and Asp178 coordinate the catalytic metal ion. The structural model was predicted by alphafold2 [15] and the figures were prepared using PyMOL [23].

Using AgDAE_5m, we have successfully established a d‐allulose production system. Moreover, we tried to improve the functions of AgDAE_5m by the construction of chimera enzyme (ApDAE and AgDAE_5m) from the data of sequence homology (Fig. 3) and the tertiary tetramer structure model with 222 symmetry (Figs 4, 5, 6) predicted by alphafold2. The improvement of enzyme function through point mutations requires consideration of cooperativity, and many experiments are required to explore various combinations of these mutations. Therefore, we selected three regions and constructed the restricted three mutants (Mutant_A, _B and _C) by transferring the sequences of ApDAE to AgDAE_5m.

#### Mutant_A (AgDAE_5m‐His13Arg/Ser245Ala)

From the sequence homology (Fig. 3) and structural similarity of the enzymes predicted by alphafold2, it was clarified that AgDAE exhibits a tetrameric structure and two loop regions [(10–14, near the N‐terminus) and (245–249, near the C‐terminus)] are located in close proximity within the three‐dimensional structure of the same molecule (Fig. 4). In the loop regions, two sites (His13 and Ser245: which are located 14 Å away from each other) in AgDAE_5m are not conserved in ApDAE. His13 and Ser245 in AgDAE_5m correspond to Arg13 and Ala245 in ApDAE (Figs 2 and 3). From the structural models of ApDAE and AgDAE_5m, it is predicted that these two sites (His13Arg/Ser245Ala) located far from the active site characterize the structures of the two loop regions. Therefore, we attempted to introduce the two loop regions (10–14 and 245–249) of ApDAE to AgDAE_5m by the two mutations (His13Arg/Ser245Ala).

### Mutant_B (AgDAE_5m‐Ala117Pro/Ala120Lys/Ala123Glu/Ala128Val/Val129Ile/Val132Ile/Asp138Gly)

In the long helix region (α4: 117–138), the sequences are not conserved between AgDAE_5m and ApDAE (Fig. 2). On α4 helix structure in ApDAE predicted by alphafold2 (Fig. 5), the structure of the main chain (Pro117), possible salt bridge (Lys120, Glu123 (Fig. 5A) and molecular packing/hydrophobic interaction (Val128, Ile129, Ile132) (Fig. 5B) appear to contribute to the stability of the helix structure without steric hindrance. Therefore, the structure of the helix of ApDAE seems to be more stable than that of AgDAE_5m. We attempted to introduce the α4 helix region (α4: 117–138) of ApDAE to that of AgDAE_5m except at position 137. The previous study [11] demonstrated that the mutation (Ser137Lys) contributes to the thermostability of the enzyme as AgDAE_5m. Similarly, ApDAE_5m exhibits increased thermostability by the mutation (Ser137Lys). Therefore, we retained Lys at position 137 for the construction of Mutant_B.

#### Mutant_C (AgDAE_5m‐Val34Ile/Pro172Gly/Ile176Val/Ser218Asn/Thr223Asn)

From the high structural similarity of two enzymes (Figs 2 and 3), positions 34, 172, 176, 218 and 223 are selected for the mutation sites for AgDAE_5m. The positions of Ser218 and Thr223 in AgDAE_5m are located at the surface between two molecules among the tetrameric structure of the enzyme (Fig. 6A) and not conserved in ApDAE (Asn218 and Asn223) (Fig. 3). By comparison between the structures (AgDAE_5m and ApDAE), the mutant Ser218Asn/Thr223Asn in AgDAE_5m (Fig. 6A) is expected to enhance the structural stability of AgDAE_5m by enhancement of the molecular packing and assembly of the tetramer structure without steric hindrance. In AgDAE_5m, Val34 is located inside the enzyme molecule (Fig. 6B). By comparing the structure of ApDAE and AgDAE_5m, we found sufficient space for replacing Ile at Val34 of AgDAE_5m (Fig. 6B). In addition, Fig. 3 also suggests the possibility of the mutation (Val34Ile). Therefore, we would expect the improvement of the thermostability of the enzyme by the enhancement of molecular packing (Val34Ile). In addition, the positions of Pro172 and Ile176 in AgDAE_5m are located at the loop and beta sheet structures inside the enzyme molecule, respectively. The proceeding His177 and Asp179 coordinates a metal ion (active site) and relates to the activity in AgDAE_5m (Fig. 6C). Therefore, it can be predicted that the loop and beta sheet structures (Fig. 6C) characterize the activity between AgDAE_5m and ApDAE. The comparison of the sequence between AgDAE_5m and ApDAE indicates that the region (170–193) is well conserved except for the 172 and 176 sites (Fig. 3). We can expect to introduce the characteristics for high activity of ApDAE to AgDAE_5m by the mutation (P172G/I176V). Therefore, we attempted to introduce the five mutations to AgDAE_5m and acquire the positive characteristics of ApDAE.

### Preparation and evaluation of the chimera enzymes

The recombinant chimera Mutant_A, _B and _C were constructed and prepared as described above. All recombinant mutants were expressed well, and their characteristics were examined (Table 1). The purity of the purified enzymes was confirmed to be over 98% by SDS/PAGE (Fig. S1). For Mutant_A, a significant improvement in specific activity was observed; however, no significant increase in the *T*_m_ value was observed. Therefore, we succeeded in construction of an improved enzyme (Mutant_A) by introducing the superior function of ApDAE without compromising thermostability. Two mutations in the two loop regions distant from the active site could be imagined to indirectly affect the structure of the active site, leading to an enhancement of enzyme activity. However, the exact mechanism underlying the enhanced specific activity remains unclear. For Mutant_B, a slight positive effect for the enzyme activity and a minor negative effect on thermostability were observed on the introduction of the α4 helix region from ApDAE to AgDAE_5m. As described above, the structure of the α4 helix region of ApDAE appears to be more stable compared to that of AgDAE_5m. Therefore, a slight steric hindrance as a result of the mutation might occur in the folding of Mutant_B. For Mutant_C, the *T*_m_ value was improved by approximately 15 °C without compromising the activity. In the case of Mutant_C, structural analysis between AgDAE_5m and ApDAE shows that the enhancement of molecular packing and/or hydrophobic interaction (Val34Ile) and the enhancement of assembly (Ser218Asn/Thr223Asn) of the tetramer structure appear to contribute the thermostability of the enzyme. The role of Pro172Gly/Ile176Val in the thermostability Mutant_C remains unclear, although these mutations did not impact enzyme activity.

In the next stage, we combined the mutants and constructed new mutant enzymes Mutant_D: Mutant_A + B + C and Mutant_E: Mutant_A + C. The characteristics of the mutants were examined (Table 1). In the case of Mutant_D, a negative effect derived from Mutant_B was also observed. We concluded that Mutant_E exhibits the best characteristics. The *T*_m_ value and specific activity of Mutant_E were improved by 18 °C and 40%, respectively, compared to ApDAE_5m (Table 1). For thermostability, an additive effect of Mutant_A and _C was observed. For specific activity, a slight synergistic effect of Mutant_A and _C was observed (Table 1). The synergistic effect might be derived from the Pro172Gly/Ile176Val mutation in Mutant_C.

**Fig. 7:**
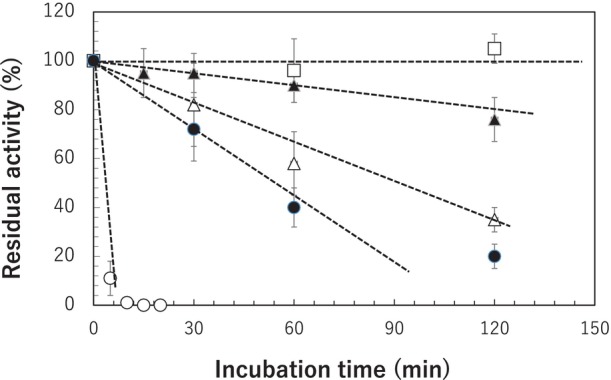
Residual activities of mutant enzymes. Enzyme samples (0.5 mg·mL^−1^) in a 50 mm sodium phosphate buffer (pH 8.0) containing 2 mm MgSO_4_ were incubated at 80 °C. ○, AgDAE; ▵, ApDAE; ●, AgDAE_5m; ▲, ApDAE_5m; □, Mutant_E). After the heat treatment, the residual activities were measured at 50 °C for 10 min under the same conditions described in the Materials and methods. The SD of three replicates is indicated by error bars.

**Fig. 8:**
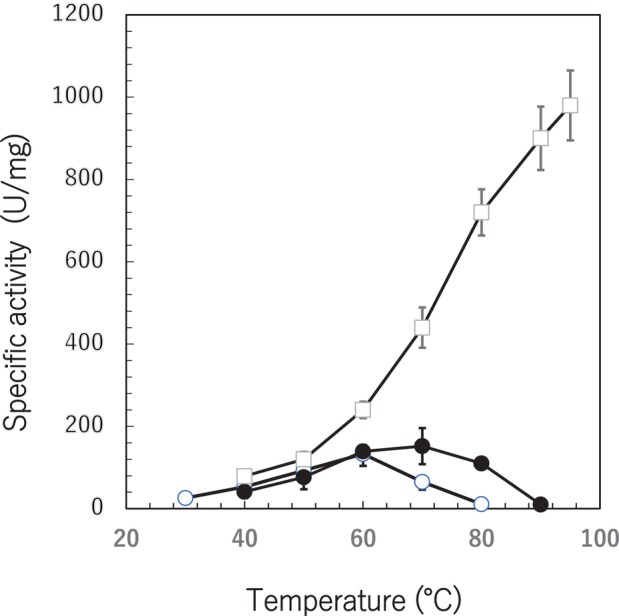
Temperature dependent activity of the mutant AgDAEs. The activity of the enzyme samples (○, AgDAE; ●, AgDAE_5m; □, Mutant_E) were measured at the designated temperatures for 10 min in a 50 mm sodium phosphate buffer (pH 8.0) containing 2 mm MgSO_4_. The activity was assayed under conditions where the reaction ratio remained within 10% by adjusting the enzyme concentration (0.5–2.0 μg·mL^−1^) and the substrate concentration is 100 mm described in the Materials and methods. The SD of three replicates is indicated by error bars.

The residual activities of the enzymes (AgDAE, AgDAE_5m, ApDAE, ApDAE_5m and Mutant_E) were examined after incubation at 80 °C and pH 8.0. The data of the residual activity exhibits the irreversible inactivation after heat treatment. However, positive results of the residual activity are required for the thermostability and application of the enzyme. As shown in Fig. 7, AgDAE lost its activity after incubation at 80 °C for 5 min. However, AgDAE_5m, ApDAE, ApDAE_5m and Mutant_E remain their activity after incubation at 80 °C. The effect was reflected in the *T*_m_ value (Table 1). It was clarified that Mutant_E exhibited highly positive effects for the residual activity assay and does not lose the activity at 80 °C. The optimum temperatures for AgDAE, AgDAE_5m and Mutant_E were examined (Fig. 8). Mutant_E exhibits an optimum temperature above 95 °C. We have no data beyond 100 °C because it is not easy to accurately measure enzyme activity around this temperature. However, the *T*_m_ value (96.08 °C) for Mutant_E (Table 1) determined by TSA suggests that the activity of Mutant_E decreases beyond 100 °C.

Enzyme kinetics were investigated with either d‐allulose and d‐fructose as the substrates at 50 °C in 50 mm sodium phosphate buffer (pH 8.0) containing 2 mm MgSO_4_ (Table 2). The values of *K*_m_ for the both substrates were slightly increased by the mutation. The *k*_cat_ value toward d‐fructose is important for the production of d‐allulose. Under the application conditions (i.e. at 50 °C and pH 8.0), the value of *k*_cat_ for Mutant_E is more than twice that of AgDAE. Furthermore, the catalytic efficiency (*k*_cat_/*K*_m_) was also improved (Table 2).

**Table 2:** Comparison of kinetic parameters. The enzyme activities were measured at 50 °C in 50 mm sodium phosphate buffer (pH 8.0) containing 2 mm MgSO_4_, described in the Materials and methods. The concentration of each enzyme is 2.0 μg·mL^−1^ and the substrate concentration ranges from 5 to 200 mm.

Fructose → Allulose			Allulose → Fructose		
*k*_cat_ (min^−1^)	*K*_m_ (mm)	*k*_cat_/*K*_m_ (min^−1^ mm ^−1^)	*k*_cat_ (min^−1^)	*K*_m_ (mm)	*k*_cat_/*K*_m_ (min^−1^ mm ^−1^)
AgDAE (1.92 ± 0.11) ×1000	21.1 ± 5.6	91.0 ± 25	(2.63 ± 0.03) ×1000	11.7 ± 0.7	225 ± 14
ApDAE (4.40 ± 0.15) ×1000	32.0 ± 3.2	138 ± 15	(4.03 ± 0.20) ×1000	9.67 ± 2	417 ± 105
Mutant_E (5.73 ± 0.33) ×1000	23.0 ± 2.5	249 ± 31	(4.15 ± 0.46) ×1000	13.5 ± 0.6	307 ± 37

## Conclusions

The improvement of enzyme activity and stability by theoretical design is continually evolving, but a complete design has not yet been developed. The results obtained in the present study indicate that the favorable characteristics of ApDAE were successfully transferred to AgDAE_5m. The chimeric construction experiments using structural information suggest that the approach to incorporate the advantages of useful enzymes is simple and effective. Currently, we cannot explain the detailed mechanism for the improvement of specific activity from the model structure predicted by alphafold2. However, the crystal structure analysis between ApDAE and AgDAE might clarify the mechanisms underlying their thermostability and activity. In the future, Mutant_E may have great potential to replace the current enzyme for d‐allulose production.

## Conflicts of interest

Kensaku Shimada, Kouhei Ohtani, Pushpa Kiran Gullapalli and Kazuhiko Ishikawa are employees of Matsutani Chemical Industry Co., Ltd.

## Peer review

The peer review history for this article is available at https://www.webofscience.com/api/gateway/wos/peer‐review/10.1002/2211‐5463.70060.

## Author contributions

KS and KO performed all experimental work, analyzed and interpreted the data, and wrote the original draft manuscript. PKG performed the development of methodology, analyzed the data and revised the manuscript. KI conceived and supervised the study, analyzed and interpreted the data, and revised the manuscript. All authors have read and approved the final version of the manuscript submitted for publication.

## Supporting information


**Fig S1.** SDS/PAGE of the purified AgDAE (WT) and its mutants.

## Data Availability

All data generated or analyzed during this study are included in this published article.
