# Supplementary material for: Construction of hyperthermostable d‐allulose 3‐epimerase from Arthrobacter globiformis M30 using the sequence information from Arthrobacter psychrolactophilus
Source: FEBS Open Bio. 2025 Jun 9;15(9):1508–19. doi: 10.1002/2211-5463.70060 (PMC12401175; doi:10.1002/2211-5463.70060)
Supplement: Supplementary file 1 — Fig S1. SDS/PAGE of the purified AgDAE (WT) and its mutants. [file FEB4-15-1508-s001.pdf]

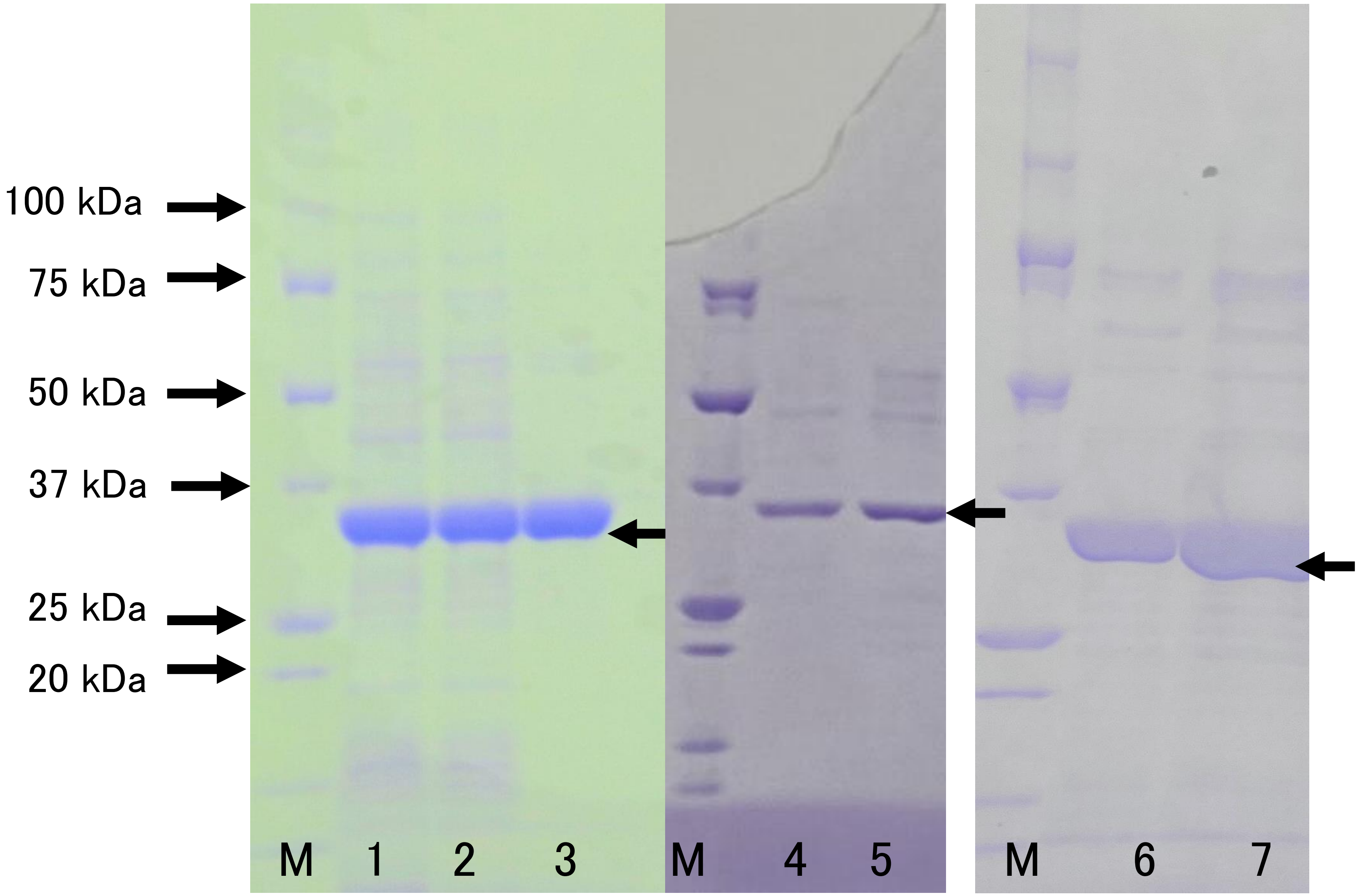

Figure S1. SDS-PAGE of the purified AgDAE (WT) and its mutants.

M, protein molecular-weight markers; lane 1, AgDAE(WT); lane 2, AgDAE_5m; lane 3, Mutant_A; lane 4, Mutant_B; lane 5, Mutant_C; lane 6, Mutant_D; lane 7, Mutant_E. The position of the purified enzymes are indicated by arrows from the right
